# A short-term concurrent training program enhances arterial stiffness and flow-mediated dilation in overweight or obese women independent of weight loss

**DOI:** 10.3389/fphys.2026.1757252

**Published:** 2026-04-27

**Authors:** Cristian Álvarez, Gabriel Rojas, David C. Andrade, Pedro Delgado-Floody, Luis Peñailillo, Alvaro N. Gurovich, Mikel Izquierdo

**Affiliations:** 1 Exercise and Rehabilitation Sciences Institute, School of Physical Therapy, Faculty of Rehabilitation Sciences, Universidad Andres Bello, Santiago, Chile; 2 Exercise Applied Physiology Laboratory, Centro de Investigación en Fisiología y Medicina de Altura (FIMEDALT), Departamento Biomédico, Facultad de Ciencias de la Salud, Universidad de Antofagasta, Antofagasta, Chile; 3 Department of Physical Education, Sports and Recreation, Universidad de La Frontera, Temuco, Chile; 4 Department of Physical Therapy and Movement Sciences, College of Health Sciences, The University of Texas at El Paso, El Paso, TX, United States; 5 Navarrabiomed, Hospital Universitario de Navarra (HUN), Universidad Publica de Navarra (UPNA), IdiSNA, Pamplona, Spain; 6 CIBER of Frailty and Healthy Aging (CIBERFES), Instituto de Salud Carlos III, Madrid, Spain

**Keywords:** concurrent training, flow-mediated dilation, obesity, overweight, pulse wave velocity

## Abstract

**Background and aims:**

Despite a wide amount of evidence regarding vascular improvements in overweight/obesity condition, little is known about similar vascular benefits from exercise training without weight loss. This study aimed to evaluate the effects of a 6-week concurrent training (CT) program on pulse wave velocity (PWV), flow-mediated dilation (FMD), and carotid intima-media thickness (cIMT) average (cIMT_av_) and maximum (cIMT_max_) in overweight/obese women who did not report weight loss. Additionally, the relationship between vascular outcomes and anthropometric and body composition measurements was examined.

**Methods:**

We conducted a secondary analysis of a clinical trial assessing the effects of CT on cardiovascular markers. Forty-three overweight/obese women (age = 42.4 ± 12.8 years; BMI = 29.7 ± 3.2 kg/m²) were divided into control (CG, *n* = 21), ‘exercise without weight loss’ (EG-NWL; *n* = 9), and ‘exercise with weight loss’ (EG-WL; *n* = 13) groups. The primary outcomes included PWV, FMD, cIMT_av_, and cIMT_max_, and the secondary outcomes included waist circumference (WC), body fat (BF), skeletal muscle mass (SMM), lean mass, and other vascular measures, reported as absolute and delta (Δ) values.

**Results:**

ΔPWV differed between CG and EG-NWL (0.2 vs. –1.2 m·s-1, *P* = 0.0002) and between CG and EG-WL (0.2 vs. –0.7 m·s-1, *P* = 0.0002). ΔFMD showed differences between CG and EG-NWL (–0.6 vs. 6.9%) and between CG and EG-WL (–0.6 vs. 4.5%). Each delta of ΔPWV, ΔFMD, ΔcIMT_av_, and ΔcIMT_max_ was significantly associated with ΔWC (*r*_s_ = 0.920; 0.912; 0.597; 0.767, *P* < 0.05), ΔBF% (*r*_s_ = 0.847; 0.791; 0.465; 0.695, *P* < 0.05), ΔSMM (*r*_s_ = 0.889; 0.566; 0.934; 0.940, *P* < 0.05).

**Conclusion:**

Overweight and obese women who did not lose weight after CT experienced a decrease in PWV and an increase in FMD. The correlations between changes in ΔPWV, ΔFMD, ΔcIMT_av_, and ΔcIMT_max_ with body composition measures suggest directions for future research.

## 1 Introduction

Overweight and obesity have been among the most pressing global health challenges for more than four decades, with prevalence continuing to rise across high- and middle-income countries. In the United States, adult obesity prevalence reached 40.3% between 2021 and 2023, disproportionately affecting women (Emmerich et al., 2024). Excess adiposity is commonly accompanied by hypertension, insulin resistance, and other cardiometabolic disorders, all of which contribute to an increased risk of cardiovascular disease (CVD) and challenge public health objectives such as the *Healthy People 2030 initiative* (U.S. Department of Health and Human Services, Office of Disease Prevention and Health Promotion, 2023). Importantly, many of these obesity-related complications manifest early as vascular dysfunction.

Obesity promotes endothelial dysfunction through chronic inflammation, oxidative stress, and lipid accumulation within the arterial wall (Jayaraj and Aburawi, 2025). This vascular impairment, characterized by reduced vasodilatory capacity and adverse arterial remodeling, represents an early and clinically meaningful marker of cardiovascular injury (Thijssen et al., 2019; Heiss et al., 2022). Accordingly, noninvasive indices such as pulse wave velocity (PWV), flow-mediated dilation (FMD), and carotid intima–media thickness (cIMT) are widely used to assess vascular function and structure, and are strongly influenced by modifiable lifestyle factors, including physical activity and diet (Pedralli et al., 2020; Cavdar et al., 2025; Photiou et al., 2025).

Body composition plays an important role in vascular health, as excess adiposity is associated with impaired endothelial function, whereas greater skeletal muscle mass has been linked to more favorable arterial properties (Santos et al., 2025; Song et al., 2025). However, growing evidence suggests that improvements in cardiovascular and metabolic health may occur independently of body weight reduction, giving rise to the “fitness versus fatness” paradigm (Farrell et al., 2002a). Indeed, experimental studies have shown that structured exercise training can improve glycemic control and vascular outcomes even in the absence of meaningful weight loss (Dekker et al., 2007).

Structured exercise training is recommended by professional organizations, including the American College of Sports Medicine (ACSM) (Pescatello et al., 2015), American Heart Association, and European Society of Cardiology (McEvoy et al., 2024). For example, ACSM guidelines emphasize that even modest weight loss (5–10%) produces clinically meaningful reductions in cardiometabolic risk (Thompson et al., 2021). Nonetheless, weight loss of <5% also confers benefits, reinforcing the importance of exercise, irrespective of weight reduction (Ozemek et al., 2025). Aerobic modalities, such as moderate-intensity continuous training (MICT) and high-intensity interval training (HIIT), reduce body weight and improve vascular outcomes. In brief (Grossman et al., 2018), demonstrated that 8 weeks of MICT or HIIT combined with diet and education led to weight losses of 3.6% and 5.3%, respectively, which increased to 4.4% and 8.6% after 16 weeks, respectively. Similarly (D’Amuri et al., 2021), found comparable weight loss effects after12 weeks of HIIT (–5.6%) and MICT (–5.5%) in adults with obesity. Resistance training (RT), in contrast, primarily promotes skeletal muscle mass (SMM) growth and may increase body mass; however, low-intensity RT has been reported to support modest weight reduction (ACSM, 2009).

Vascular adaptations to exercise are particularly relevant beyond weight outcomes. Eight weeks of HIIT decreased PWV by –0.7 m·s^−1^ and increased FMD by +3.6%, despite negligible weight loss (Sawyer et al., 2016). In contrast, 12 weeks of MICT did not alter cIMT in healthy men and was associated with only minimal reductions in body mass (Tanaka et al., 2002). Concurrent training (CT), which combines MICT and RT modalities, has been proposed to maximize both metabolic and vascular benefits. Huang et al. showed that 8 weeks of CT plus diet and education yielded greater weight loss (–7.7%) than CT alone (–3.3%) (Huang et al., 2023). Pedralli et al. demonstrated that in overweight and obese adults with hypertension, 8 weeks of CT induced weight loss (–1.5%) and improved FMD (+6.8%), outperforming MICT (–1.5%; +3.2%) and RT alone (–0.5%; +4.0%) (Pedralli et al., 2020). Although these findings highlight CT as an effective strategy for improving vascular outcomes, few studies have investigated CT protocols combining HIIT and RT or their impact on PWV, FMD, and cIMT in adults who do not lose weight. Molecular adaptations to HIIT include enhanced oxidative capacity (Hood et al., 2011) which may accelerate fat metabolism, whereas RT is critical for maintaining SMM and supporting mitochondrial biogenesis (Ruple et al., 2021). Thus, CT combining HIIT and RT (CT_HIIT+RT_) may have synergistic effects on vascular health and metabolic function, particularly in individual’s resistance to weight loss. We and others have previously reported improvements in secondary vascular indices, such as the augmentation index and ankle–brachial index, following CT (Álvarez et al., 2024a). However, the effects of CT_HIIT+RT_ on primary vascular outcomes in overweight and obese adults without weight loss remain unknown.

Accordingly, the present study aimed to examine the effects of a 6-week CT_HIIT+RT_ program combining HIIT and RT on vascular function, assessed by PWV, FMD, cIMT average, and cIMT maximum, in overweight and obese adult women who did not achieve weight loss. A secondary aim was to evaluate the associations between vascular adaptations and changes in anthropometric and body composition parameters. We hypothesized that CT_HIIT+RT_ would improve vascular outcomes independently of reductions in weight.

## 2 Materials and methods

### 2.1 Population and study design

This study is a secondary analysis of our VASCU-HEALTH study, which is a randomized controlled clinical trial developed initially in 60 adult participants from a university community (Álvarez et al., 2023, 2024; Álvarez et al., 2024; Álvarez et al., 2024b). Following a personal interview, a screening was conducted for each participant to assess their health information and eligibility for the study. All participants were randomly assigned to either the control (non-exercise) group or the exercise group and agreed to undergo a 6-week CT_HIIT+RT_ exercise program that included both HIIT and RT, conducted three times a week. However, for this study, we included only (*n* = 43) adult women with an established overweight or obesity condition (BMI between 25 and 35 kg/m^2^). Thus, the participants data were grouped into controls (CG), exercise group with ‘weight loss’ (EG-WLR), and exercise group ‘without weight loss’ (EG-NWL). The study was registered at ClinicalTrials.gov (NCT05710653) and approved by the Institutional Review Board of the Bioethics Committee of Universidad Andres Bello (Approval 026/2022 of September 22^nd^). The study followed the Declaration of Helsinki for human studies, and all participants provided written consent before participating in the study.

The VASCU-HEALTH study had the following inclusion criteria: *i*) normal BMI [18.5 to 24.9 kg/m^2^] or overweight/obese based on BMI [that is, BMI 25.0 to 35.0 kg/m^2^]; *ii*) elevated fasting glucose or type 2 diabetes mellitus (T2DM, *i.e*. treated with pharmacotherapy); and *iii*) residing in areas near the exercise facility to promote good adherence. The exclusion criteria were as follows: *i*) history of abnormal cardiac rhythm (*i.e.* ECG history), diagnosis of other cardiovascular conditions/history other than hypertension, and vasculopathy; *ii*) history of uncontrolled stage 3 hypertension or hypertensive crisis; *iii*) diabetes complications such as varicose ulcers and nephropathies; *iv*) SMM abnormalities (*e.g*., knee or hip arthrosis and muscle pain); *v*) use of weight loss treatment/pharmacotherapy or being active in exercise training programs (or within the past three months); *vi*) use of other pharmacotherapy that can influence body weight loss; and *vii*) to be physically under international guidelines (World Health Organization, 2020). However, in addition to this secondary study, the *viii*) normal nutritional state (BMI 18.5 to 24.9 kg/m^2^) was determined as an ‘exclusion criterion’.

**Figure 1:**
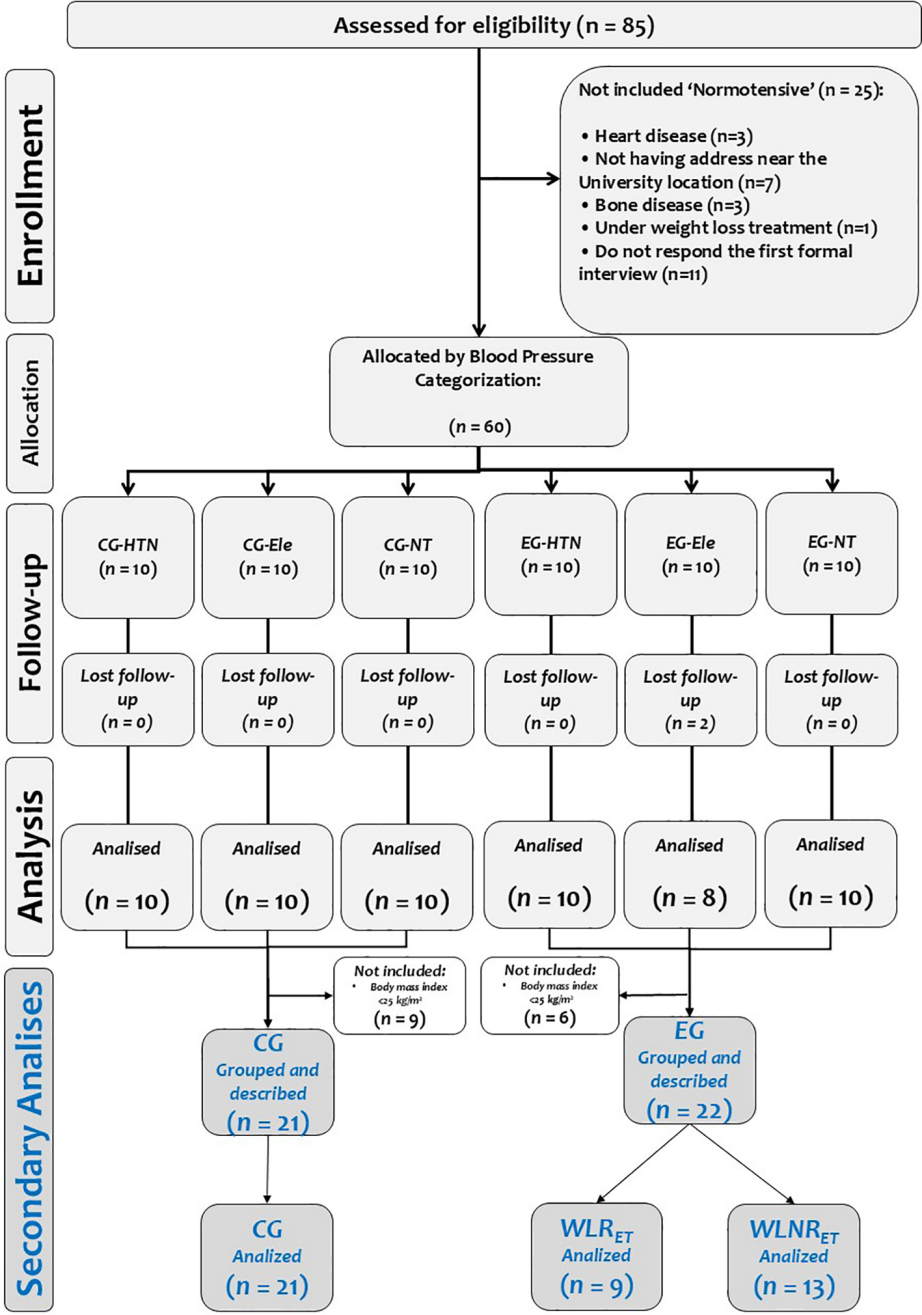
Study design. The gray boxes denote the original studies. The blue boxes represent the current secondary study. The groups were described as follows: (CG-HTN) Control group hypertensive. (CG-Ele) Control group with elevated blood pressure. (CG-NT) Control group normotensive. (EG-HTN) Exercise group hypertensive (EG-Ele). The exercise group showed elevated blood pressure. (EG-NT) Exercise group normotensive. (CG) control group. (EG) exercise group. (EG-NWL) No weight loss in exercise training. (EG-WL) Weight loss during exercise training.

The sample size to this secondary study was calculated according to the delta changes observed in the systolic blood pressure (ρSBP = −19.0 mmHg; SD = 9.0 mmHg) from a previous study by our research team (Álvarez et al., 2024a). A statistical power analysis revealed that a total of six participants per group would yield a power of 80% at a 0.05 alpha level. Thus, the final sample size included only overweight/obese (BMI>25.0 kg/m^2^) participants, grouped into control (CG; *n* = 21) and exercising (EG; *n* = 22) groups; from here, EG were grouped/categorized into participants who exercised with ‘no weight loss’ (EG-NWL; *n* = 9) and exercise ‘weight loss’ (EG-WL; *n* = 13). More details of the original and current secondary analyses of the study intervention can be seen in the CONSORT study design (**Figure 1**).

### 2.2 Weight loss and no weight loss categorization

To differentiate participants who lost weight after the exercise program from those who did not, we used the technical error of measurement (TE) calculated from previous studies by our team (TE: 0.5 kg) (Álvarez et al., 2018). Therefore, as shown in (**Figure 1**), only overweight/obese participants from the (EG, *n* = 22) were re-categorized as exercise ‘no weight loss’ (EG-NWL, *n* = 9) or exercise weight loss (EG-WL, *n* = 13) if they did not lose or did lose 0.5 kg after the 6-week CT_HIIT+RT_ program.

### 2.3 Blood pressure measurement

Blood pressure was measured following the American Heart Association 2018 guidelines, defined as follows: systolic (SBP)/diastolic (DBP) blood pressure <120/80 mmHg for normotension, elevated blood pressure 120 to 129/80 mmHg, stage 1 hypertension 130 to 139/80 to 89 mmHg, and stage 2 hypertension ≥140/90 mmHg (Whelton et al., 2018). We previously suggested avoiding coffee and food intake at least one hour before the lab visit. Two measurements were taken in the left arm (by a cuff in the brachial artery) after a 10-minute seated rest using an automatic monitor (OMRON™ HEM 7114, Kyoto, Japan). Using post-test minus pre-test absolute values, delta changes (Δ) were obtained for each SBP (ΔSBP) and DBP (ΔDBP). From here, the delta of pulse pressure (ΔPP) and mean arterial pressure (ΔMAP) were calculated using both ΔSBP and ΔDBP data. We also measured the delta of systolic blood pressure of the ankle (ΔSBP_ank_). To do this, each patient remained in a resting position for 5 minutes and using the same equipment for brachial blood pressure (OMRON™ HEM 7114, Kyoto, Japan), we obtained SBP_ank_.

### 2.4 Anthropometric and body composition outcomes

We used the post-test *minus* pre-test absolute values, to obtain delta changes (Δ) in anthropometric (Δweight, kg; ΔBMI, kg/m^2^; Δwaist circumference, cm) and body composition outcomes (Δbody fat, %; ΔSMM, kg; and ΔLean mass, kg). All outcomes were measured using a bio-impedanciometer (HBF-514C OMRON™ Healthcare Inc., Lake Forest, IL, United States). Height (m) was measured using a stadiometer (Health o Meter™ Professional, Sunbeam Products, Inc., Chicago, IL, United States). Waist circumference and delta (ΔWC, cm) were measured using an inextensible tape (SECA™, United States) and using the World Health Organization procedure at the umbilicus level and after the exhalation phase of respiration (World Health Organization, 2008). On the other hand, although it is not reported in this study, we used the (Alberti et al., 2009) cutoff value of >80 cm as a risk factor for the specific group of South American women. BMI and delta changes (ΔBMI, kg/m^2^) were calculated using weight divided by the square of the height (WHO, 2000). All anthropometric measurements were performed by the same trained professional, both in the pre- and post-intervention assessments. These measurements were developed in the morning between 9:00 and 12:00 hours at the Exercise and Rehabilitation Sciences Institute (ICER) of the Universidad Andres Bello.

### 2.5 Arterial stiffness (main outcome)

Arterial stiffness was measured using the PWV post-test *minus* the PWV pre-test of absolute values to obtain delta changes in PWV (ΔPWV). This outcome was measured in the brachial artery using oscillometric pressure traces from the brachial artery in the upper left arm (measured in m·s^-1^) with an arteriograph device after a 20-minute rest in the supine position (Arteriograph, Tensiomed™, BUD, Hungary). Data analysis was conducted using Arteriograph Software v.1.9.9.2. The blood pressure assessment algorithm of this device has been validated (Ring et al., 2014). PWV values exceeding ≥10 m·s^-1^ indicate elevated arterial stiffness, which correlates with an increased cardiovascular risk (Mancia et al., 2013).

### 2.6 Flow-mediated dilation of the brachial artery (main outcome)

After 10 minutes of rest, all participants were evaluated for their blood pressure (OMRON™ HEM 7114, Kyoto, Japan). All explorations were performed using ultrasound equipment (General Electric™, Model LOGIQ-E PRO, Milwaukee, United States) with a 7−12 MHz linear–array transducer. FMD calculation was developed in six phases. First, each participant was placed in the supine position for 20 min on a medical bed, with the arm in abduction at 90°. A blood pressure cuff (Riester model ri-san™, Jungingen, Germany) was positioned on the forearm, and the ultrasound transducer (*i.e.* 1–3 cm proximal to the antecubital fossa on a longitudinal plane) was installed in the brachial artery using an adjustable mechanical arm precision holder (EDI™, Progetti e Sviluppo, Italy) to maintain the transducer position and increase evaluator standardization. Thus, the baseline diameter of the brachial artery (D_base_) images (*i.e.*, in B mode and also Doppler function) was obtained (*i.e*. during 30 to 60 sec) and stored in the ‘off-mode’ to develop the measurement (*i.e.* 2 to 4 diameter measurements and the average was registered). Second, the blood pressure cuff of the forearm was inflated at 50 mmHg above the resting SBP, initiating a 5-minute forearm occlusion to induce reactive hyperaemia (Thijssen et al., 2019). Third, immediately after cuff deflation, every 10 s for 120 s (12 images) were obtained and stored in the ‘off-mode’ to determine the peak diameter measurement (D_peak_) (*i.e.* 2 to 4 diameter measurements and the average was registered) in each image. Fourth; FMD obtained from the formulae: $\text{FMD }(\%)=\frac{[(\text{Dpeak}\;-D\text{base})]*100}{\text{Dbase}}$ A FMD >6.6% proposed by the European Society of Hypertension and the European Society of Cardiology (Mancia et al., 2013) were considered acceptable cut-off points for normal vasodilation. Reliability was estimated using intraclass correlation coefficients based on four baseline measurements of 0.91 for baseline diameter and 0.83 for FMD (Ramírez-Vélez et al., 2019). More details about the FMD procedure have been previously reported (Álvarez et al., 2024a).

### 2.7 Carotid intima-media thickness (main outcome)

To measure cIMT_av_ and cIMT_max_, we used an ultrasound imaging 7–12 MHz linear-array transducer (General Electric™, Model LOGIQ-E PRO, Milwaukee, United States). The subjects were placed in the supine position for 20 min. After carotid bulb identification, an image was obtained in ‘B mode’ for the right longitudinal orientation of the common carotid artery using an automatic ultrasound function that detects both cIMT_av_ and cIMT_max_. The scan was focused 1 cm from the carotid bifurcation on the far wall. The ultrasound software recorded the images, which were subsequently analyzed. All measurements were recorded at the end-diastolic stage (Coll and Feinstein, 2008). Given that cIMT_av_ >0.9 mm has been used as a previous cut-off point to denote high cardiovascular risk, we used this value for our cIMT_max_ outcome, following the European Society of Hypertension and European Society of Cardiology (Mancia et al., 2013).

### 2.8 Secondary cardiovascular parameters (secondary outcomes)

Using the same Arteriograph equipment in the brachial artery after a 20-minute rest in a supine position (Arteriograph, Tensiomed™, BUD, Hungary) and the Arteriograph Software (v.1.9.9.2, BUD, Hungary) we obtained the secondary vascular outcomes; heart rate at supine position (HR_sup_, beats/min), augmentation index of the brachial artery (Aix, %), ankle brachial index (ABI), ejection duration (ED, m·s^-1^), and return time (RetT, m·s^-1^) (Morales et al., 2015), where each delta change (Δ) was calculated. To acquire the ABI outcome from the equipment, the SBP of the left ankle (SBP_ank_) was measured in each subject using an automatic blood pressure monitor (OMRON™ HEM 7114, Kyoto, Japan) before starting each measurement, prior to PWV analysis. All main and secondary vascular outcomes were blinded by operator.

### 2.9 Concurrent training of high-intensity interval plus resistance training

The participants exercised three times per week, with 48–72 h between sessions, for six consecutive weeks. The program was conducted from the afternoon to the early evening. Each CT_HIIT+RT_ session included HIIT using stationary bikes, followed by RT using free weights. For HIIT, each participant performed five 1-minute sets between 80% and 100% of HR_peak_ with a resting period until HR returned to ≤70% HR_peak_ controlled individually by heart rate monitors (Model A370, Polar™, Finland) on upright stationary bicycles (Impulse™, model PS 300, Sparta, Chile) (Olea et al., 2017). After a 3 to 5-min cool-down, the RT section started, and participants completed 1-min sets of resistant exercises as follows: 1) biceps curl [× 2 sets], 2) shoulder press [× 2 sets], and 3) back exercise [× 1 set], performed at 20 to 50% of 1RM, and with a resting period between sets until a modified Borg scale rating of 1 to 3, out of 10 points. The total workout session last for 30–40 minutes and both HIIT and RT exercise followed the American College of Sports Medicine progression recommendations (Thompson et al., 2021). To regulate HIIT and RT intensity, subjects received bike-load session-by-session accommodations (80% to 100% of HR_peak_ for HIIT) and RT at a perceived muscle exertion of 7 to 10 points (modified Borg scale), ensuring each participant maintained an appropriate individual exercise intensity. One week before starting the 6-week CT, the participants performed a volitional progressive Astrand test to determine HR_peak_. They developed the one-maximum repetition test (1RM) in which the best of three dynamic strength attempts (*i.e.*, concentric/eccentric movement) was registered, and this information has been previously reported (Álvarez et al., 2024). In the first 3 weeks, the exercise order was HIIT+RT, and in the last 3 weeks, it was RT+HIIT. According to our laboratory protocol, each participant was evaluated for their blood pressure before and after the exercise sessions. In addition to the ~30 min of only exercise, we included 10 min before exercise and 10 min after exercise for blood pressure control, estimating a total of ~50 min time-investment per session in each participant.

### 2.10 Statistical analysis

Data are presented as mean ± standard deviation (SD). Normality and homoscedasticity assumptions were tested using the Shapiro-Wilk test. For outcomes with normal distribution, 2-way ANOVA (Groups × time) was applied, while for outcomes without normal distribution, the Kruskal-Wallis test with Dunn’s *post hoc* test was used. Cohen’s *d* effect size (<0.2 = ‘negligible’, 0.2-0.49 = ‘small’, 0.5-0.79 = ‘moderate’, ≥0.8 = ‘large’) for interactions that showed statistically significant (Cohen, 2013). Each delta (Δ), representing pre-post changes in the main and secondary outcomes, was calculated for the CG, EG-WL, and EG-NWL groups using the absolute difference between post-test and pre-test values. One-way ANOVA was used to compare the CG, EG-WL, and EG-NWL groups. To test the association among the primary outcomes (ΔPWV, ΔFMD, ΔcIMT_av_, and ΔcIMT_max_) with anthropometric, body composition, and cardiovascular outcomes, simple linear regression (to parametric) with R^2^ prediction percentage, or the *Rho* Spearman nonparametric correlation (*r*_s_ =) was applied. The main outcomes (ΔPWV, ΔFMD, ΔcIMT_av_, and ΔcIMT_max_) associated with anthropometric/body composition (ΔWC, ΔBF, ΔSMM, and ΔLM) are shown in Figures, while secondary cardiovascular associations are shown in Table. Statistical analyses were performed using Prism 8.0 software Graph Pad, San Diego, CA, United States). The alpha level was fixed at (*P ≤* 0.05) for all statistical significance.

## 3 Results

### 3.1 Baseline general sample

The planned adherence was 18 sessions (100%); the general EG group was (88.8%), with the EG-NWL: 17 sessions (94.4%) and EG-WL: 15 sessions (83.3%) adherence. General sample characteristics of the CG and EG showed no baseline differences among the outcomes studied (**Table 1**).

**Table 1:** General physiological characteristics of anthropometry, body composition, cardiovascular and hemodynamic vascular of overweight/obesity adult women participants of 6 weeks of concurrent training of high-intensity interval plus resistance training.

Outcome	Time	CG	EG	Between-group CG *vs*. EG
*Morbility* (*n* = )		21	22	
Hypertensive (*n* = )		7	7	
Elevated blood pressure *(n* = )		6	7	
Normotensive *(n* = )		8	8	
Diabetes *(n* = )		0	1	
*Anthropometry * Age (y)	Pre	40.7 ± 12.6	44.2 ± 13.1	*P* = 0.370^&^; 0.01
Height (m)	Pre	1.63 ± 0.09	1.64 ± 0.08	*P* = 0.677^&^; 0.004
Weight (kg)	Pre	80.2 ± 12.9	79.3 ± 12.4	*P* = 0.811; 0.001
	Post	80.2 ± 12.8	78.8 ± 12.4	
	*P*_value;_ *_d_*	*P* = 0.867; 0.001	*P* = 0.082; 0.13	
	Δ	0	–0.5	*P* = 0.198^&^; 0.04
Body mass index (kg/m^2^)	Pre	30.1 ± 3.4	29.4 ± 3.0	*P* = 0.432; 0.01
	Post	30.1 ± 3.4	29.2 ± 2.9	*P* = 0.803^Ω^
	*P*_value;_ *_d_*	*P* = 0.303; 0.05	*P* = 0.437; 0.02	
	Δ	0.0	–0.2	*P* = 0.329^&^; 0.02
Waist circumference (cm)	Pre	99.9 ± 7.7	100.3 ± 7.4	*P* = 0.859; 0.0007
	Post	100.0 ± 7.3	97.1 ± 8.1	*P* = 0.817^Ω^
	*P*_value;_ *_d_*	*P* = 0.778; 0.005	***P* < 0.0001; 0.61*^d^***	
	Δ	+0.1	–3.2	***P* < 0.0001^&^; 0.34**
Body composition				
Body fat (%)	Pre	39.2 ± 8.8	40.0 ± 6.0	*P* = 0.734; 0.002
	Post	39.7 ± 8.7	37.9 ± 6.6	*P* = 0.841^Ω^
	*P*_value;_ *_d_*	*P* = 0.434; 0.12	***P* = 0.003; 0.25**	
	Δ	+0.5	–2.1	***P=*0.004^&^; 0.18**
Skeletal muscle mass (%)	Pre	28.9 ± 7.1	26.0 ± 3.7	*P* = 0.104; 0.06
	Post	29.0 ± 7.2	27.4 ± 4.1	*P* = 0.238^Ω^
	*P*_value;_ *_d_*	*P* = 0.612; 0.05	***P=*0.001; 0.36**	
	Δ	+0.1	+1.4	***P=*0.007^&^; 0.16**
Lean mass (kg)	Pre	48.7 ± 11.2	47.8 ± 10.4	*P* = 0.771; 0.002
	Post	48.4 ± 11.3	49.1 ± 10.4	*P* = 0.908^Ω^
	*P*_value;_ *_d_*	*P* = 0.409; 0.17	***P=*0.007; 0.21**	
	Δ	–0.3	+1.3	***P=*0.007^&^; 0.16**
Cardiovascular secondary outcomes				
Heart rate rest (sit) (b/min)	Pre	76.2 ± 7.3	78.7 ± 11.2	*P* = 0.551; 0.008
	Post	76.3 ± 6.0	75.1 ± 7.6	*P* = 0.855^Ω^
	*P*_value;_ *_d_*	*P* = 0.968; 0.0006	***P* = 0.004; 0.20**	
	Δ	+0.1	–3.6	***P=*0.032^&^; 0.10**
Heart rate rest (supine) (b/min)	Pre	73.1 ± 8.0	73.0 ± 8.0	*P* = 0.967; 0.008
	Post	73.4 ± 7.7	70.6 ± 9.6	*P* = 0.575^Ω^
	*P*_value;_ *_d_*	*P* = 0.845; 0.04	***P* = 0.047; 0.04**	
	Δ	+0.3	–2.4	*P* = 0.924^&^; 0.0002
Heart rate peak (b/min)	Pre	139.4 ± 51.2	129.6 ± 55.9	*P* = 0.552; 0.005
	Post	138.0 ± 49.6	136.4 ± 58.0	*P* = 0.638^Ω^
	*P*_value;_ *_d_*	*P* = 0.326; 0.09	***P<*0.0001; 0.43**	
	Δ	–1.4	+6.8	***P=*0.0002^&^; 0.29**
Augmentation index (%)	Pre	–21.1 ± 18.2	–15.0 ± 24.6	*P* = 0.366; 0.01
	Post	–16.9 ± 21.2	–23.2 ± 20.5	*P* = 0.875^Ω^
	*P*_value;_ *_d_*	*P* = 0.216; 0.08	***P* = 0.015; 0.22**	
	Δ	+4.2	+8.2	***P* = 0.007^&^; 0.13**
Ankle-Brachial Index	Pre	1.16 ± 0.08	1.13 ± 0.09	*P* = 0.243; 0.03
	Post	1.16 ± 0.08	1.18 ± 0.10	*P* = 0.741^Ω^
	*P*_value;_ *_d_*	*P* = 0.847; 0.008	***P<*0.0001; 0.45**	
	Δ	0	+0.05	***P* = 0.007^&^; 0.28**
Ejection duration (m·s^-1^)	Pre	304.5 ± 21.0	310.8 ± 13.3	*P* = 0.247; 0.03
	Post	306.2 ± 23.1	308.2 ± 17.0	*P* = 0.455^Ω^
	*P*_value;_ *_d_*	*P* = 0.532; 0.10	*P* = 0.456; 0.02	
	Δ	+1.7	–2.6	*P* = 0.259; 0.03
Return time (m·s^-1^)	Pre	132.9 ± 23.2	121.4 ± 21.1	*P* = 0.096; 0.06
	Post	131.6 ± 23.1	128.5 ± 20.7	*P* = 0.165^Ω^
	*P*_value;_ *_d_*	*P* = 0.517; 0.22	***P<*0.001; 0.26**	
	Δ	–1.3	+7.1	***P* = 0.003^&^; 0.18**

Data are shown as mean and ± standard deviation. Groups are described as (CG) Control group and (EG) Exercise group. Statistical changes are shown as:.

(Ω) Groups x time comparisons analyzed by two-way ANOVA.

(^&^) Analyzed by unpaired *t*-test at *P* < 0.05.

(*d*) Denotes Cohen d effect size at *P* < 0.05 (Bold values indicate moderate or significant clinical effects).

All bold values denote significant statistical changes/differences at *P ≤* 0.05.

### 3.2 Pre-post changes in general sample

Within-group analyses showed that the EG exhibited delta changes (Δ) of a reduced waist circumference (ΔWC –3.2 cm, *P* < 0.0001; *d* 0.61), body fat (ΔBF –2.1%, *P* < 0.0001; *d* 0.25), increased skeletal muscle mass (ΔSMM +1.4%, *P* = 0.001; *d* 0.36), increased lean mass (ΔLM +1.3 kg, *P* = 0.007; *d* 0.21), reduced heart rate at rest (sit) (ΔHRR –3.6 b/min, *P* = 0.004; *d* 0.20), increased heart rate peak (ΔHR_peak_ +6.8 b/min, *P* = 0.043; *d* 0.43), increased augmentation index (ΔAix +8.2%, *P* = 0.015; *d* 0.22), increased ankle brachial index (ΔABI +0.05, *P* < 0.0001; *d* 0.45), and increased return time (ΔRetT +7.1 m·s^-1^, *P* < 0.001; *d* 0.26) (**Table 1**).

Between-group analyses revealed significant differences between the CG and EG in delta outcomes waist circumference (ΔWC 0.1 vs. –3.2 cm, *P* < 0.0001; *d* 0.34), body fat (ΔBF +0.5 vs. –2.1%, *P* = 0.004; *d* 0.18), skeletal muscle mass (ΔSMM 0.1 vs. –1.4%, *P* = 0.007; *d* 0.16), lean mass (ΔLM –0.3 vs. +1.3 kg, *P* = 0.007; *d* 0.16), heart rate rest (sit) (ΔHRR 0.1 vs. –3.6 b/min, *P* = 0.032; *d* 0.10), heart rate peak (ΔHR_peak_ –1.4 vs. 6.8 b/min, *P* = 0.0002; *d* 0.29), augmentation index (ΔAix 4.2 vs. 8.2%, *P* = 0.007; *d* 0.16), and return time (ΔRetT –1.3 vs. 7.1 m·s^-1^, *P* = 0.003; *d* 0.18), (**Table 1**).

### 3.3 Training-induced changes in vascular parameters (primary outcomes)

**Figure 2:**
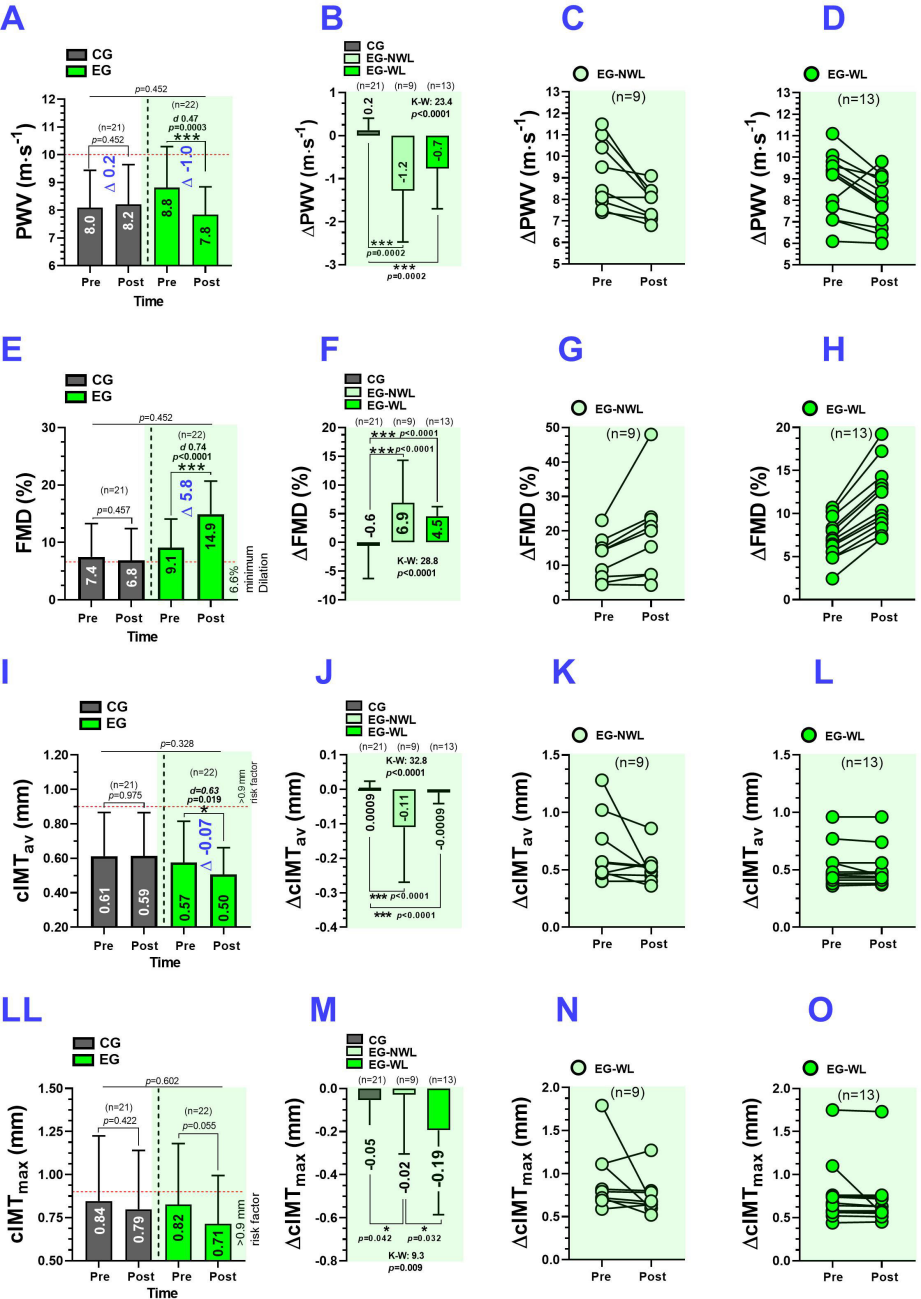
Pulse wave velocity, flow-mediated dilation, carotid intima-media thickness average, and maximum carotid intima-media thickness after 6-weeks of concurrent training in adults with cardiovascular risk factors. Groups are described as: (CG) control group, (EG) exercise group, (EG-NWL) exercise group without weight loss, and (EG-WL) exercise group with weight loss. Outcomes were described as: (PWV) pulse wave velocity, (FMD) flow-mediated dilation, (cIMT_av_) carotid intima-media thickness average, and (cIMT_max_) carotid intima-media thickness maximum. **(A, E, I, L)** Show the information of each CG and EG grouped in the general sample by absolute values of PWV, FMD, cIMT_av,_ and cIMT_max,_ respectively. **(B, F, J, M)** show the information grouped by EG-NWL and EG-WL in delta changes (pre-post) values of PWV, FMD, cIMT_av_ and cIMT_max_ respectively. **(C, G, K, N)** show the information of only EG-NWL participants in individual values of PWV, FMD, cIMT_av,_ and cIMT_max,_ respectively. **(D, H, L, O)** show the information of only EG-WL subjects in individual values of PWV, FMD, cIMT_av,_ and cIMT_max,_ respectively. (-–) Intermittent red line denote cutt-off point of low and high cardiovascular risk. (Δ) denotes delta pre-post changes. **(A, E, I, LL)**; (*) denotes significant differences between pre-and post-test at *P* < 0.05. (***) Denotes significant differences between pre-and post-test at *P* < 0.0001. (*d*) Denotes Cohen d effect size at *P* < 0.05 (Bold values indicate moderate or large clinical effects). **(B, F, J, M)**; (*) Denotes significant differences between pre-and post-test at *P* < 0.05. (***) Denotes significant differences between pre-and post-test at *P* < 0.0001. (K–W) Data were analyzed using the Kruskal-Wallis nonparametric test at *P* < 0.05, and Dunn’s nonparametric *post-hoc* test for multiple comparisons at *P* < 0.05. All bold values denote significant statistical changes/differences at *P ≤* 0.05.

The CG showed remained unchanged in PWV (8.0 ± 1.3 to 8.2± 1.4 m·s^-1^, *P* = 0.452) (**Figure 2A**). The EG showed significant decrease in absolute PWV (8.8 ± 1.4 to 7.8 ± 1.0 m·s^-1^, *P* = 0.0003, (**Figure 2A**), and ΔPWV–1.0 m·s^-1^, *d* 0.47). There were significant differences in delta of ΔPWV between CG vs. EG-NWL and EG-WL (0.2 vs. –1.2 m·s^-1^, *P* = 0.0002 and 0.2 vs. –0.7 m·s^-1^, *P* = 0.0002, respectively) (**Figure 2B**). Individual data showed that all EG-NWL participants had a decrease in ΔPWV (**Figure 2C**). In contrast, in the EG-WL group (*n* = 1), the participants reported a worse response (**Figure 2D**).

The absolute values of FMD did not change after CT intervention in the CG; however, the EG reported a significant increase (9.1 ± 4.9 to 14.9 ± 5.7%, *P* < 0.0001, ΔFMD +5.8%, *d* 0.74) (**Figure 2E**). There were significant differences in the delta changes of ΔFMD between the CG and EG-NWL and EG-EG-WL (–0.6 vs. +6.9%, *P* < 0.0001 and +0.6 vs. +4.5%, *P* < 0.0001, respectively) (**Figure 2F**). Individual data showed that only one subject in the EG-NWL group (*n* = 1) showed a decrease in ΔFMD (**Figure 2G**), whereas all participants in the EG-WL group showed an increase in FMD (**Figure 2H**). cIMT_av_ did not elicit significant modifications in absolute data in the CG; however, cIMT_av_ decreased significantly in the EG (0.57 ± 0.11 to 0.50 ± 0.9 m·s^-1^, *d* 0.63, *P* = 0.019, (**Figure 2I**). There were significant differences in delta changes of ΔcIMT_av_ between the CG and EG-NWL (+0.0009 vs. –0.11 mm, *P* < 0.0001) and between the CG and EG-WL (+0.0009 vs. –0.0009 mm, *P* < 0.0001) (**Figure 2J**). Individual data showed that in the EG-NWL group (*n* = 1), ΔcIMT_av_ increased (**Figure 2K**), whereas in the EG-WL group (*n* = 3), ΔcIMT_av_ increased (**Figure 2L**). The absolute cIMT_max_ did not show any modifications in the CG and EG groups (**Figure 2LL**). There were significant differences in delta of ΔcIMT_max_ between the CG and EG-NWL (–0.05 vs. –0.02 mm, *P* = 0.042) and between the EG-NWL and EG-WL groups (–0.02 vs. –0.19 mm, *P* = 0.032) (**Figure 2M**). Individual data indicate that the EG-NWL group (*n* = 2) showed an increase in ΔcIMT_max_ (**Figure 2N**), whereas the EG-WL group (*n* = 1) showed no change in this outcome (**Figure 2O**).

### 3.4 Training-induced changes in blood pressure (secondary outcomes)

**Figure 3:**
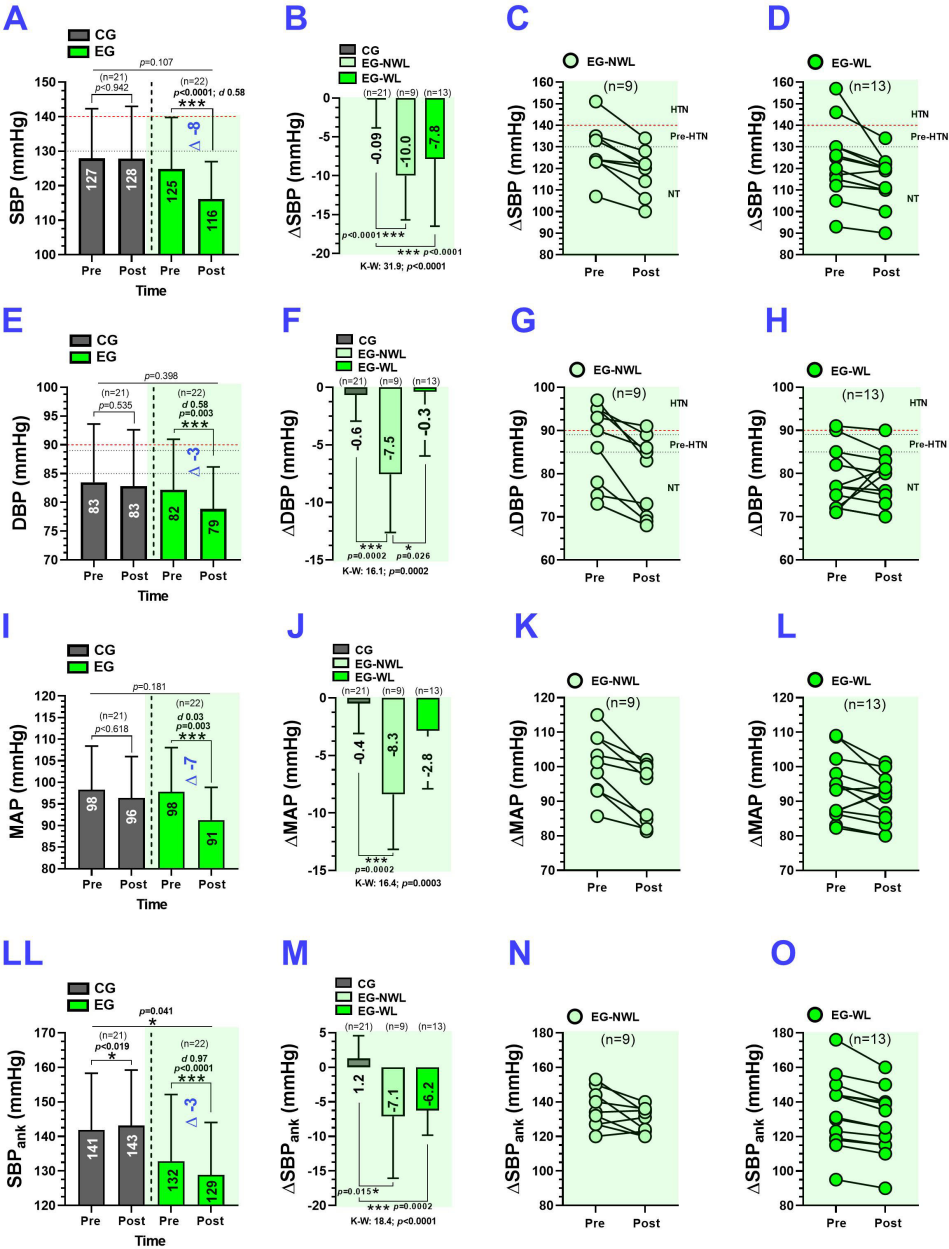
Systolic blood pressure, diastolic blood pressure, mean arterial pressure and systolic blood pressure of the ankle after 6-weeks of concurrent training in adults with cardiovascular risk factors. Groups are described as; (CG) Control group, (EG) Exercise group, (EG-NWL) Exercise-group no weight loss and (EG-WL) Exercise-group weight loss. Outcomes are described as; (SBP) Systolic blood pressure, (DBP) diastolic blood pressure, (MAP) Mean arterial pressure and (SBP_ank_) Systolic blood pressure of the ankle. Panels **(A, E, I, LL)** show the information of each CG and EG in absolute values of SBP, DBP, MAP and SBP_ank,_ respectively. **(B, F, J, M)** show the information of each EG-NWL and EG-WL in delta changes (pre-post) values of SBP, DBP, MAP and SBP_ank,_ respectively. **(C, G, K, N)** Show the information of only EG-NWL subjects in individual values of SBP, DBP, MAP and SBP_ank,_ respectively. **(D, H, L, O)** Show the information of only EG-WL subjects in individual values of SBP, DBP, MAP and SBP_ank,_ respectively. (-–) Intermittent red line denotes cut-off point of low and high cardiovascular risk. (Δ) Denotes delta pre-post changes. **(A, E, I, L)**; (*) Denotes significant differences between pre vs. post-test at *P* < 0.05. (***) Denotes significant differences between pre vs. post-test at *P* < 0.0001. (*d*) Denotes Cohen d effect size at *P* < 0.05 (Bold values indicate moderate or large clinical effects). **(B, F, J, M)**; (*) Denotes significant differences between pre vs. post-test at *P* < 0.05. (***) Denotes significant differences between pre vs. post-test at *P* < 0.0001. (K–W) Data analyzed by Kruskall-Wallis nonparametric test at *P* < 0.05, and Dunn’s nonparametric *post-hoc* for multiple comparisons at *P* < 0.05. All bold values denote significant statistical changes/differences at *P ≤* 0.05.

The CG showed no changes in the absolute SBP. In contrast, the EG showed significant reductions (125 ± 15 to 116 ± 11 mmHg, *P* < 0.0001, ΔSBP –8.0 mmHg, *d* 0.58) (**Figure 3A**). There were significant differences in ΔSBP between the CG and EG-NWL (–0.09 vs. –10.0 mmHg, *P* < 0.0001) and between the CG and EG-WL groups (–0.09 vs. –7.8 mmHg, *P* < 0.0001) (**Figure 3B**). Individual data showed that all EG-NWL participants had a decrease in ΔSBP (**Figure 3C**), while the EG-WL group had only one participant (*n* = 1) with increased ΔSBP (**Figure 3D**). The CG showed no changes in absolute DBP, while the EG showed significant reductions (82 ± 9 to 79 ± 7 mmHg, *P* = 0.003, ΔDBP –3 mmHg, *d* 0.58) (**Figure 3E**). There were significant differences in delta ΔDBP between the CG and EG-NWL (–0.6 vs. –7.5 mmHg, *P* = 0.0002) and between the EG-NWL and EG-WL groups (–7.5 vs. –0.3 mmHg, *P* = 0.026) (**Figure 3F**). Individual data showed that all EG-NWL participants had decreased DBP (**Figure 3G**). In contrast, the EG-WL group (*n* = 3) showed an increase in DBP (**Figure 3H**). The CG showed no changes in the absolute values of MAP. At the same time, the EG elicited significant reductions in MAP (98 ± 10 to 91 ± 10 mmHg, *P* = 0.003, ΔMAP –7 mmHg, *d* 0.03) (**Figure 3I**). There were significant differences in ΔMAP between the CG and EG-NWL groups (–0.4 vs. –8.3 mmHg, *P* = 0.0002) (**Figure 3J**). Individual data showed that all EG-NWL subjects had decreased MAP (**Figure 3K**), whereas the EG-WL group (*n* = 3) showed an increase in ΔMAP (**Figure 3L**). The CG showed significant increases in absolute SBP_ank_ (141 ± 17 to 143 ± 17, *P* = 0.019), while the EG group showed significant reductions in SBP_ank_ (132 ± 19 to 129 ± 15 mmHg, *P* < 0.0001, ΔSBP_ank_ –3 mmHg, *d* 0.97) (**Figure 3LL**). There were significant differences in delta ΔSBP_ank_ between the CG and EG-NWL (1.2 vs. –7.1 mmHg, *P* = 0.015) and between the CG and EG-WL groups (1.2 vs. –6.2 mmHg, *P* = 0.0002) (**Figure 3M**). Individual data showed that all EG-NWL (*n* = 3) participants increased ΔSBP_ank_ (**Figure 3N**), whereas the EG-WL group showed a decrease in ΔSBP_ank_ (**Figure 3O**).

### 3.5 Training-induced changes in anthropometric, body composition, and secondary cardiovascular parameters in weight loss and no weight loss groups (secondary outcomes)

Within-group analyses showed that both EG-NWL and EG-WL showed significant delta changes in waist circumference (ΔWC –3.1 cm, *P* = 0.019, *d* 0.51; –3.1 cm, *P* = 0.0002, *d* 0.70) and HR_peak_ (ΔHR_peak_ +10.3 b/min, *P* = 0.033, *d* 0.45; +6.2 b/min, *P* < 0.0001, *d* 0.84) (**Table 2**). Additionally, the EG-NWL showed a significant increase in weight (ΔWeight +0.7 kg, *P* = 0.046, *d* 0.41) and increased return time (ΔRT +11.7 m·s^-1^, *P* = 0.039, *d* 0.43) (**Table 2**). The EG-NWL also reported changes in body fat (ΔBF, –2.8%, *P* = 0.013, *d* 0.41), skeletal muscle mass (ΔSMM, +1.9%, *P* = 0.05, *d* 0.49), lean mass (ΔLM, +1.4 kg, *P* = 0.002, *d* 0.54), resting heart rate (HRR, –3.9 beats/min, *P* = 0.0006, *d* 0.67), and ankle brachial index (ΔABI, +0.05, *P* < 0.0001, *d* 0.73) (**Table 2**). Considering those outcomes with similar benefits, no significant differences between groups were detected in waist circumference and HR_peak_ (**Table 2**).

**Table 2:** Anthropometric, body composition, and secondary cardiovascular outcomes of adult women participants of 6 weeks of concurrent training high-intensity interval plus resistance training categorized in exercise no weight loss and exercise weight loss after intervention.

Outcomes	Time	EG-NWL	EG-WL	Between-group *P*value
*Morbility* (*n* = )	Pre	9	13	
Hypertensive *n* = (%)	Pre	4	3	
Elevated blood pressure *n* = (%)	Pre	4	3	
Normotensive *n* = (%)	Pre	1	7	
Diabetes *n* = (%)	Pre	0	1	
Age (y)	Pre	44.6 ± 15.1	44.0 ± 12.1	*P* = 0.910^&^
Height (m)	Pre	1.64 ± 0.09	1.64 ± 0.08	*P* = 0.923^&^
*Anthropometry*				
Weight (kg)	Pre	80.3 ± 15.3	78.6 ± 10.5	*P* = 0.762^&^
	Post	81.0 ± 15.2	77.2 ± 10.5	
	*P*_value; d_	***P* = 0.046; 0.41**	***P* < 0.0001; 0.76*^d^***	
	Δ	+0.7	–1.4	***P* < 0.0001^&^**
Body mass index (kg/m^2^)	Pre	29.8 ± 3.8	30.1 ± 2.3	*P* = 0.296^&^
	Post	30.1 ± 3.1	28.7 ± 2.9	
	*P*_value;_ *_d_*	*P* = 0.953; 0.0004	*P* = 0.064; 0.25	
	Δ	+0.3	–1.4	*P* = 0.322^&^
Waist circumference (cm)	Pre	99.7 ± 9.1	100.7 ± 6.4	*P* = 0.785^&^
	Post	96.6 ± 10.4	97.5 ± 6.4	
	*P*_value;_ *_d_*	***P* = 0.019; 0.51*^d^***	***P* = 0.0002; 0.70*^d^***	
	Δ	–3.1	–3.1	*P* = 0.083^&^
Body composition				
Body fat (%)	Pre	40.2 ± 6.9	41.9 ± 5.7	*P* = 0.521
	Post	39.9 ± 5.7	39,1 ± 5.9	
	*P*_value;_ *_d_*	*P* = 0.909; 0.0008	***P* = 0.013; 0.41**	
	Δ	–0.3	–2.8	*P* = 0.062^&^
Skeletal muscle mass (%)	Pre	25.7 ± 3.9	26.3 ± 4.6	*P* = 0.774^&^
	Post	26.2 ± 3.7	28.2 ± 3.7	
	*P*_value;_ *_d_*	*P* = 0.206; 0.19	***P* = 0.005; 0.49**	
	Δ	+0.5	+1.9	*P* = 0.098^&^
Lean mass (kg)	Pre	48.2 ± 12.0	47.5 ± 12.2	*P* = 0.879^&^
	Post	48.7 ± 9.6	48.9 ± 9.4	
	*P*_value;_ *_d_*	*P* = 0.945; 0.0003	***P* = 0.002; 0.54*^d^***	
	Δ	+0.5	+1.4	*P* = 0.395^&^
Cardiovascular/Vascular				
Heart rate rest (sit) (b/min)	Pre	83.7 ± 15.0	80.2 ± 11.6	*P* = 0.377^&^
	Post	82.3 ± 13.3	77.6 ± 11.5	
	*P*_value;_ *_d_*	*P* = 0.292; 0.13	***P* = 0.0006; 0.67*^d^***	
	Δ	–3.0	–3.9	*P* = 0.380^&^
Heart rate rest (supine) (b/min)	Pre	70.2 ± 7.6	75.0 ± 8.0	*P* = 0.171^&^
	Post	66.7 ± 11.4	73.3 ± 7.4	
	*P*_value;_ *_d_*	*P* = 0.350; 0.10	*P* = 0.220; 0.12	
	Δ	–3.5	–1.7	*P* = 0.804^&^
Heart rate peak (b/min)	Pre	157.1 ± 22.5	144.9 ± 17.5	*P* = 0.201^&^
	Post	167.4 ± 18.0	151.1 ± 16.3	
	*P*_value;_ *_d_*	*P* = 0.033; 0.45	***P<*0.0001; 0.84*^d^***	
	Δ	+10.3	+6.2	*P* = 0.268^&^
Augmentation index (%)	Pre	–11.7 ± 29.7	–17.3 ± 23.0	*P* = 0.615^&^
	Post	–20.9 ± 23.0	–24.8 ± 19.3	
	*P*_value;_ *_d_*	*P* = 0.196; 0.03	*P* = 0.065; 0.25	
	Δ	9.2	7.5	*P* = 0.663^&^
Ankle-Brachial Index	Pre	1.10 ± 0.06	1.15 ± 0.11	*P* = 0.615^&^
	Post	1.16 ± 0.12	1.20 ± 0.10	
	*P*_value;_ *_d_*	*P* = 0.106; 0.29	***P<*0.0001; 0.73*^d^***	
	Δ	+0.06	+0.05	*P* = 0.962^&^
Ejection duration (m·s^-1^)	Pre	313.9 ± 12.9	308.7 ± 13.7	*P* = 0.383^&^
	Post	311.7 ± 23.7	305.8 ± 10.7	
	*P*_value;_ *_d_*	*P* = 0.789; 0.009	*P* = 0.309; 0.08	
	Δ	–2.2	–2.9	*P* = 0.925^&^
Return time (m·s^-1^)	Pre	118.6 ± 23.2	123.4 ± 20.2	*P* = 0.610^&^
	Post	130.3 ± 20.9	127.2 ± 21.2	
	*P*_value;_ *_d_*	***P* = 0.039; 0.43**	*P* = 0.137; 0.10	
	Δ	+11.7	+3.8	*P* = 0.137^&^

Data are shown as mean and ± standard deviation. Groups are described as (CG) Control group and (EG) Exercise group. Statistical changes are shown as;.

(Ω) Groups x time comparisons analyzed by two-way ANOVA.

(^&^) Analyzed by unpaired *t*-test at *P* < 0.05.

(*d*) Denotes Cohen *d* effect size at *P* < 0.05 (Bold values indicate moderate or large clinical effects).

All bold values denote significant statistical changes/differences at *P ≤* 0.05.

### 3.6 Association between vascular with anthropometric/body composition outcomes

**Figure 4:**
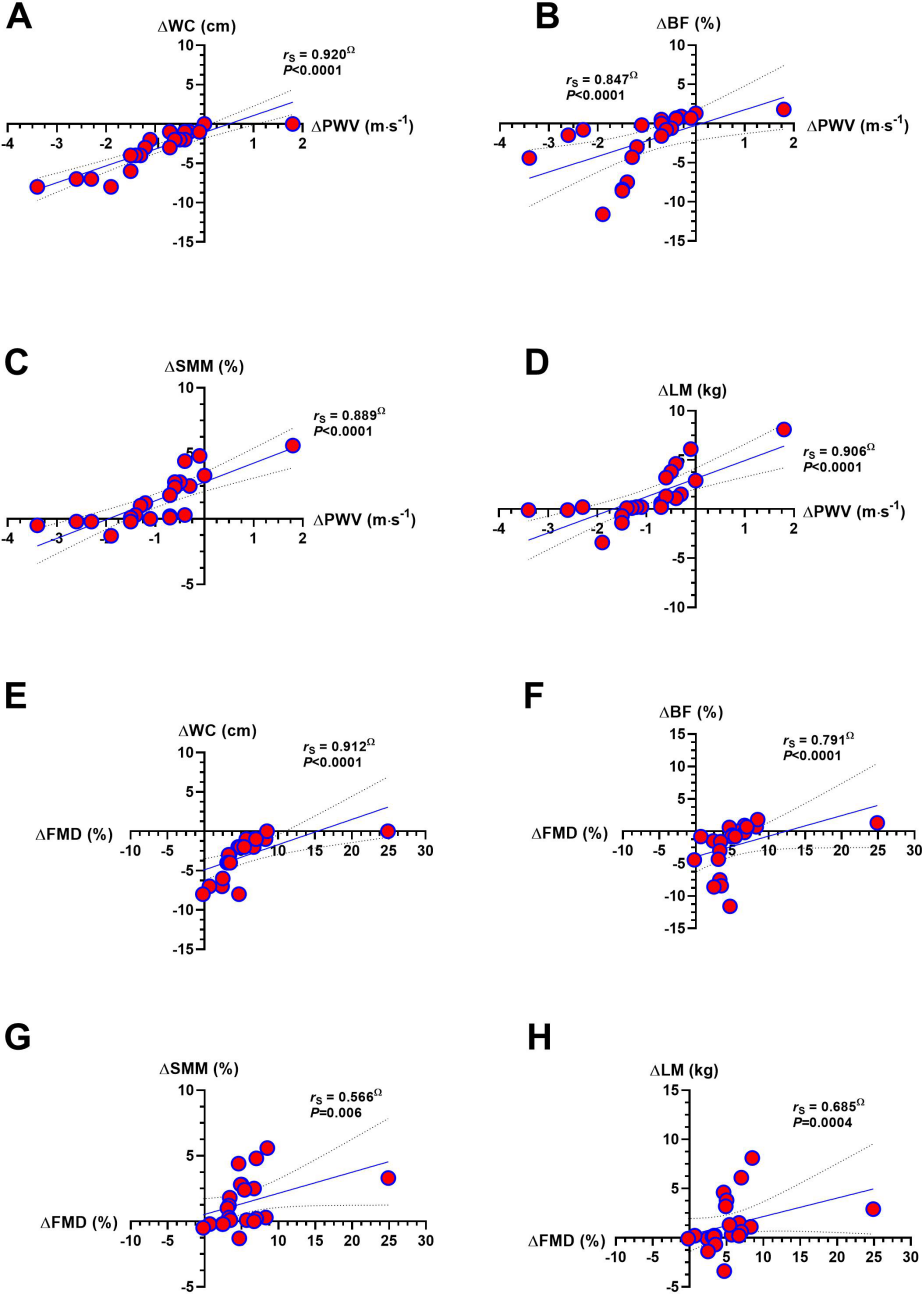
Association between the delta changes of pulse wave velocity (ΔPWV) with; delta waist circumference (ΔPWV, **A**), delta body fat percentage (ΔBF, **B**), delta skeletal muscle mass percentage (ΔSMM, **C**), delta lean mass (ΔLM, **D**), and association between delta of FMD (ΔFMD) with delta waist circumference (ΔWC, **E**), delta body fat percentage (ΔBF, **F**), delta of skeletal muscle mass percentage (ΔSMM, **G**), and with delta lean mass (ΔLM, **H**), reported after 6-weeks of concurrent training in adults with cardiovascular risk factors. Outcomes are described as follows: (ΔPWV) delta pulse wave velocity, (ΔWC) delta waist circumference, (ΔSMM) delta skeletal muscle mass, (ΔLM) delta lean mass, and (ΔFMD) delta flow-mediated dilation. (^Ω^) denotes the Spearman’s rho correlation nonparametric test. The intermittent parallels lines denote (95%CI) of the slope. Bold *P values* denote significant associations between ΔPWV and each Δ outcome correlated.

The delta of ΔPWV was significantly associated with delta outcomes ΔWC (*r*_s_ = 0.920, *P* < 0.0001), ΔBF% (*r*_s_ = 0.847, *P* < 0.0001), ΔSMM (*r*_s_ = 0.889, *P* < 0.0001), and ΔLM kg (*r*_s_ = 0.906, *P* < 0.0001) (**Figures 4A, D**. The delta of ΔFMD was significantly associated with delta outcomes ΔWC (*r*_s_ = 0.912, *P* < 0.0001), ΔBF% (*r*_s_ = 0.791, *P* < 0.0001), ΔSMM (*r*_s_ = 0.566, *P* = 0.006), and ΔLM kg (*r*_s_ = 0.685, *P* = 0.0004) (**Figures 4E, H**). The delta of ΔcIMT_av_ was significantly associated with delta outcomes of ΔWC (*r*_s_ = 0.597, *P* = 0.003), ΔBF% (*r*_s_ = 0.465, *P* = 0.028), and ΔSMM% (*r*_s_ = 0.934, *P* < 0.0001) (**Figures 4A, D**). Similarly, the delta of ΔcIMT_max_ was significantly associated with delta outcomes of ΔWC (*r*_s_ = 0.767, *P* < 0.0001), ΔBF% (*r*_s_ = 0.695, *P* = 0.0003), and ΔSMM% (*r*_s_ = 0.940, *P* < 0.0001), and with delta of ΔLM (*r*_s_ = 0.829, *P* < 0.0001) (**Figures 4E, H**).

### 3.7 Association between vascular with secondary anthropometric and cardiovascular outcomes

After 6-weeks of CT intervention, the delta of ΔPWV was significantly associated with delta of ΔBMI (R^2^ 0.433 [43.3%], *P* = 0.0009), ΔSBP (*r*_s_ = 0.932, *P* < 0.0001), ΔDBP (*r*_s_ = 0.748, *P* < 0.0001), ΔMAP (*r*_s_ = 0.850, *P* < 0.0001), and ΔABI (*r*_s_ = 0.511, *P* = 0.014) (**Table 3**). The delta of ΔFMD was significantly associated with delta of Δ weight (*r*_s_ = 0.552, *P* = 0.012), ΔBMI (*r*_s_ = 0.697, *P* = 0.0003), ΔSBP (*r*_s_ = 0.646, *P* = 0.011), ΔMAP (*r*_s_ = 0.748, *P* < 0.0001), and ΔABI (*r*_s_ = 0.445, *P* = 0.037) (**Table 3**). The delta of ΔcIMT_av_ was significantly associated with delta of ΔSBP (*r*_s_ = 0.911, *P* < 0.0001), ΔDBP (*r*_s_ = 0.918, *P* < 0.0001), and ΔMAP (*r*_s_ = 0.922, *P* < 0.0001), (**Table 3**). The delta of ΔcIMT_max_ was significantly associated with delta of ΔBMI (*r*_s_ = 0.613, *P* = 0.002), ΔSBP (*r*_s_ = 0.960, *P* < 0.0001), ΔDBP (*r*_s_ = 0.845, *P* < 0.0001), ΔMAP (*r*_s_ = 0.892, *P* < 0.0001), and ΔABI (*r*_s_ = –0.444, *P* = 0.038), (**Table 3**).

**Table 3:** Associations between primary vascular outcomes ΔPWV, ΔFMD, ΔcIMT_av_ and ΔcIMT_max_ with anthropometric and secondary cardiovascular outcomes in overweight/obesity woman participants of 6 weeks of concurrent training.

	*Parametric or nonparametric correlation test*	
Outcomes	*P_value_; R^2^; Equation*	*r_s_ =; P_value_*
*Anthropometric*		
ΔWeight (kg) – ΔPWV (m·s^-1^)		*r*_s_ = 0.416; *P* = 0.054^Ω^
ΔWeight (kg) – ΔFMD (%)		***r*_s_ = 0.552; *P* = 0.012^Ω^**
ΔWeight (kg) – ΔcIMT_av_ (mm)		*r*_s_ = –0.065; *P* = 0.772
ΔWeight (kg) – ΔcIMT_max_ (mm)		*r*_s_ = 0.247; *P* = 0.265
ΔBMI (kg/m^2^) – ΔPWV (m·s^-1^)	*P* = 0.0009; R^2^ 0.433; Y = 1.160*X+1.623^†^	
ΔBMI (kg/m^2^) – ΔFMD (%)		***r*_s_ = 0.697; *P* = 0.0003^Ω^**
ΔBMI (kg/m^2^) – ΔcIMT_av_ (mm)		*r*_s_ = 0.405; *P* = 0.061^Ω^
ΔBMI (kg/m^2^) – ΔcIMT_max_ (mm)		***r*_s_ = 0.613; *P* = 0.002^Ω^**
*Secondary cardiovascular*		
ΔSBP (mmHg) – ΔPWV (m·s^-1^)		***r*_s_ = 0.932; *P<*0.0001^Ω^**
ΔSBP (mmHg) – ΔFMD (%)		***r*_s_ = 0.646; *P* = 0.011^Ω^**
ΔSBP (mmHg) – ΔcIMT_av_ (mm)		***r*_s_ = 0.911; *P<*0.0001^Ω^**
ΔSBP (mmHg) – ΔcIMT_max_ (mm)		***r*_s_ = 0.960; *P<*0.0001^Ω^**
ΔDBP (mmHg) – ΔPWV (m·s^-1^)		***r*_s_ = 0.748; *P<*0.0001^Ω^**
ΔDBP (mmHg) – ΔFMD (%)		*r*_s_ = 0.415; *P=*0.054^Ω^
ΔDBP (mmHg) – ΔcIMT_av_ (mm)		***r*_s_ = 0.918; *P<*0.0001^Ω^**
ΔDBP (mmHg) – ΔcIMT_max_ (mm)		***r*_s_ = 0.845; *P<*0.0001^Ω^**
ΔMAP (mmHg) – ΔPWV (m·s^-1^)	*P* = 0.0009; R^2^ 0.760; Y = 4.601*X–0.6426^†^	
ΔMAP (mmHg) – ΔFMD (%)		***r*_s_ = 0.748; *P<*0.0001^Ω^**
ΔMAP (mmHg) – ΔcIMT_av_ (mm)		***r*_s_ = 0.922; *P<*0.0001^Ω^**
ΔMAP (mmHg) – ΔcIMT_max_ (mm)		***r*_s_ = 0.892; *P<*0.0001^Ω^**
ΔSBP_ank_ (mmHg) – ΔPWV (m·s^-1^)	*P* = 0.936; R^2^ 0.0003	
ΔSBP_ank_ (mmHg) – ΔFMD (%)		*r*_s_ = 0.015; *P=*0.945^Ω^
ΔSBP_ank_ (mmHg) – ΔcIMT_av_ (mm)		*r*_s_ = 0.358; *P=*0.101^Ω^
ΔSBP_ank_ (mmHg) – ΔcIMT_max_ (mm)		*r*_s_ = 0.369; *P=*0.090^Ω^
ΔHR at rest (b/min) – ΔPWV (m·s^-1^)		*r*_s_ = 0.132; *P=*0.558^Ω^
ΔHR at rest (b/min) – ΔFMD (%)		*r*_s_ = 0.060; *P=*0.787^Ω^
ΔHR at rest (b/min) – ΔcIMT_av_ (mm)		*r*_s_ = 0.101; *P=*0.977^Ω^
ΔHR at rest (b/min) – ΔcIMT_max_ (mm)		*r*_s_ = –0.202; *P=*0.365^Ω^
ΔHR_peak_ (b/min) – ΔPWV (m·s^-1^)		*r*_s_ = 0.217; *P=*0.330^Ω^
ΔHR_peak_ (b/min) – ΔFMD (%)		*r*_s_ = 0.275; *P=*0.215^Ω^
ΔHR_peak_ (b/min) – ΔcIMT_av_ (mm)		*r*_s_ = –0.108; *P=*0.630^Ω^
ΔHR_peak_ (b/min) – ΔcIMT_max_ (mm)		*r*_s_ = –0.067; *P=*0.763^Ω^
ΔHR_sup_ (b/min) – ΔPWV (m·s^-1^)	*P* = 0.735; R^2^ 0.005	
ΔHR_sup_ (b/min) – ΔFMD (%)		*r*_s_ = 0.045; *P=*0.839^Ω^
ΔHR_sup_ (b/min) – ΔcIMT_av_ (mm)		*r*_s_ = 0.101; *P=*0.652^Ω^
ΔHR_sup_ (b/min) – ΔcIMT_max_ (mm)		*r*_s_ = –0.066; *P=*0.768^Ω^
ΔAix (%) – ΔPWV (m·s^-1^)		*r*_s_ = 0.294; *P=*0.183^Ω^
ΔAix (%) – ΔFMD (%)		*r*_s_ = 0.299; *P=*0.175^Ω^
ΔAix (%) – ΔcIMT_av_ (mm)		*r*_s_ = –0.252; *P=*0.257^Ω^
ΔAix (%) – ΔcIMT_max_ (mm)		*r*_s_ = –0.168; *P=*0.452^Ω^
ΔABI – ΔPWV (m·s^-1^)		*r*_s_ = 0.511; *P=*0.014^Ω^
ΔABI – ΔFMD (%)		*r*_s_ = 0.445; *P=*0.037^Ω^
ΔABI – ΔcIMT_av_ (mm)		*r*_s_ = –0.392; *P=*0.070^Ω^
ΔABI – ΔcIMT_max_ (mm)		*r*_s_ = –0.444; *P=*0.038^Ω^
ΔEjection duration – ΔPWV (m·s^-1^)		*r*_s_ = 0.256; *P=*0.249^Ω^
ΔEjection duration – ΔFMD (%)		*r*_s_ = 0.188; *P=*0.401^Ω^
ΔEjection duration – ΔcIMT_av_ (mm)		*r*_s_ = 0.137; *P=*0.542^Ω^
ΔEjection duration – ΔcIMT_max_ (mm)		*r*_s_ = 0.297; *P=*0.178^Ω^
ΔReturn time (m·s^-1^) – ΔPWV (m·s^-1^)		*r*_s_ = 0.744; *P=*0.740^Ω^
ΔReturn time (m·s^-1^) – ΔFMD (%)		*r*_s_ = 0.011; *P=*0.961^Ω^
ΔReturn time (m·s^-1^) – ΔcIMT_av_ (mm)		*r*_s_ = –0.182; *P=*0.417^Ω^
ΔReturn time (m·s^-1^) – ΔcIMT_max_ (mm)		*r*_s_ = –0.134; *P=*0.551^Ω^

(*r*_S_ = ) Nonparametric *Rho* Spearman correlation coefficient. (Δ) Delta pre-post difference. Outcomes are described as; (PWV) Pulse wave velocity, (FMD) Flow mediated dilation, (cIMT_av_) Carotid intima-media thickness average, (cIMT_max_) Carotid intima-media thickness maximum. (HR) Heart rate, (HR_peak_) Heart rate peak during cardio-respiratory and volitional exercise Astrand test in bike, (HR_sup_) Hear rate rest at supine position. (ΔBMI) Delta body mass index, (ΔSBP) Delta systolic blood pressure, (ΔDBP) Delta diastolic blood pressure, (ΔMAP) Delta mean arterial pressure, (ΔSBP_ank_) Delta systolic blood pressure of the ankle, (ΔAix) Delta augmentation index of the brachial artery, (ΔABI) Delta ankle-brachial index. (^<s>†</s>^) Analyzed by simple linear regression test. (^Ω^) Analyzed by *Rho* Spearman nonparametric correlation (*r*_s_ = ). Bold values denote significant correlations at *P ≤* 0.05.

## 4 Discussion

### 4.1 Main findings

This study examined the effects of a 6-week CT program, which combined HIIT with low-intensity RT, on vascular function in overweight and obese women, focusing on participants who did not lose weight. Three main findings emerged: (i) CT induced ‘modest’ weight loss in 59% (*n* = 13) of participants; (ii) both weight-loss (EG-WL) and no-weight-loss (EG-NWL) groups demonstrated vascular improvements compared with controls non-exercisers; and (iii) changes in vascular markers (ΔPWV, ΔFMD, ΔcIMT_av_, and ΔcIMT_max_) were associated with changes in anthropometric, body composition, and secondary cardiovascular parameters.

Current ACSM guidelines recommend a 5–10% reduction in body weight for individuals with obesity to achieve meaningful cardiometabolic benefits (Jakicic et al., 2024). Although participants in our study achieved a modest –1.7% reduction, this aligns with prior evidence that even minimal weight loss can yield health benefits (Sawyer et al., 2016; Grossman et al., 2018; Pedralli et al., 2020; Huang et al., 2023). For instance (Sawyer et al., 2016), observed negligible weight loss after 8 weeks of MICT or HIIT alone in insulin-resistant women, whereas (Huang et al., 2023) reported greater reductions (–7.7%) when CT was combined with dietary education. Therefore, our findings highlight that short-term exercise interventions without dietary support may initiate weight reduction, but longer programs (12–16 weeks) or multimodal interventions are needed for clinically meaningful losses (Grossman et al., 2018).

On the other hand, although the primary aim of the present study was not to investigate weight loss per se, but rather to use the identification of weight-loss responders to describe their post-exercise vascular behavior following concurrent training (CT), the literature has described different factors responsible for weight reduction after exercise. For example, the EG-WL group reported a significant body-weight reduction of −1.4 kg, which represented a smaller magnitude (−1.7%) compared with the recommendations from specialized institutions to perceive cardiometabolic health benefits through physical exercise (Jakicic et al., 2024). In this context, and under these considerations, even in the absence of statistically significant differences, the EG-NWL group also exhibited changes in body composition that are consistent with the observed reduction of −3.1 cm in waist circumference in both groups. These findings may reflect underlying physiological mechanisms (i.e., not directly assessed or reported) that could have favored weight loss in some participants. Additionally, the EG-NWL group, increased muscle mass by +0.5% and fat-free mass by +0.5 kg, which is relatively consistent with the observed increase of +0.7 kg in total body weight. Therefore, the reduction of −3.1 cm in waist circumference are highly consistent with the literature, which suggests that maintaining or increasing trained muscle mass enhances mitochondrial number and density, thereby promoting fat oxidation (Zhao and Wu, 2023). However, the purpose of the present study was precisely to test whether vascular benefits were present in the subgroup of individuals who lost weight and to describe them, with the magnitude of weight loss not playing a central role in our analysis. This approach is supported by evidence showing that individuals with higher adiposity (i.e., overweight/obesity) tend to lose weight more rapidly than those with normal weight. Thus, given the similarities at baseline between the EG-NWL and EG-WL groups in terms of initial body weight and BMI, differences in body composition changes were observed exclusively in the variables ΔSMM (+1.9%) and ΔLM (+1.4%) in favor of the EG-WL group, whereas anthropometric outcomes showed a similar reduction in waist circumference (−3.1 cm). A review and meta-analysis by (Bellicha et al., 2021) reported that exercise interventions based on MICT, HIIT, and RT lasting at least 12 weeks induce weight loss ranging from −1.5 to −3.5 kg, and that RT-related losses in LM range from −0.4 to −1.3 kg values that fall within the EG-WL range group observed. Another relevant point to consider is the improvement observed in ΔWC reduction and the non-significant decrease in body fat in the EG-NWL group, despite the absence of significant changes in body weight and BMI. In this regard, both groups (EG-NWL and EG-WL) performed the same exercise protocol CT_HIIT+RT_, in which the RT component was prescribed using relatively low loads (20–60% of one-repetition maximum).

Although it is traditionally accepted that higher RT loads (i.e., ≥80% of one-repetition maximum) are associated with greater muscle hypertrophy, recent evidence suggests that increases in muscle mass may depend more strongly on internal physiological mechanisms, particularly muscle protein synthesis. In this context (Lees et al., 2026), reported that muscle hypertrophy responses were comparable when using both high- and low-load RT protocols. On the other hand, because both groups also performed HIIT, it is important to note that the waist circumference measurement is a surrogate marker of abdominal adipose tissue. HIIT is characterized by promoting elevated catecholamine responses (i.e., adrenaline and noradrenaline), which have a high affinity for stimulating lipolysis in abdominal adipose tissue (Boutcher, 2010). This is largely due to the greater density of β-adrenergic receptors in this fat depot (Zhu et al., 2024). Therefore, these physiological mechanisms may partly explain the reduction in waist circumference observed in the EG-NWL group, even in the absence of significant changes in body weight or BMI. Future improvements to elucidate potential mechanisms underlying greater or lesser reductions in body weight include the use of more robust body composition assessment methods, such as dual-energy X-ray absorptiometry, and reporting segmental changes in upper- and lower-limb muscle mass and adiposity, as well as weekly dietary intake and other factors that may differentially increase energy expenditure in favor of one group over the other. Other potential causes may also include hormonal mechanisms (e.g., beta adrenergic pathways and appetite regulation) (Boutcher, 2010), sleep, stress (Theodorakis et al., 2024), and the post-exercise oxygen consumption (BaiQuan et al., 2025) which could, to some extent, favor reductions in weight and should be explored in future studies.

### 4.2 Vascular changes

Regarding cIMT_av_ and cIMT_max_, these outcomes were less responsive within the 6-week period, but only EG-NWL reduced cIMT_av_ in comparison with the CG, and no changes were observed in cIMT_max_ in this group. Previous studies have suggested that longer interventions (≥12 months) may be required to elicit structural changes in carotid wall thickness (Magalhães et al., 2019). Given the differences in sensitivity between cIMT_av_ and cIMT_max_, future studies should incorporate both measures, alongside hemodynamic and biomarker assessments, to better elucidate the underlying mechanisms about the sensitivity of both outcomes to exercise interventions. Similarly (Ferreira et al., 2003), studied 118 adolescents aged 13–16 years old, and found that increased cardiorespiratory fitness due to increased physical activity until 36 years of age was associated with decreased arterial stiffness and reduced cIMT_av_ in adulthood. Thus, despite unchanged weight, anthropometric or body composition parameters in our ‘no weight loss’ group, vascular parameters related to structural changes could also be improved under overweight/obesity conditions by presumably cardiorespiratory fitness improvements that we have not included at all in the present study. Dekker et al. after 12 weeks of MICT (60 min [five times/week]) without weight loss results, reported that overweight/obesity T2DM adults patients decreased fasting glucose –9 mg/dL, ΔWC –2.7 cm and visceral fat in –7 kg (Dekker et al., 2007). Following this, both the EG-NWL and EG-WL groups showed an increase in HR_peak_, which was a direct exercise effect independent of the weight loss condition (**Table 2**). Unfortunately, HR_max_ was not associated with cIMT_av_ or cIMT_max_ (**Table 3**), indicating a cause-effect relationship that warrants investigation in future studies.

Furthermore, although it is difficult to establish definitive causality, the reduction in arterial stiffness observed in the EG-NWL group (ΔPWV −1.2 m·s^−1^) may be more strongly associated with the HIIT rather than RT exercise component of the CT_HIIT+RT_ program. Previous evidence supports this notion. For instance, a 6-month HIIT intervention consisting of 4-minute cycling intervals at 90% of maximal HR interspersed with 3-minute active recovery periods at 70% reduced PWV from 8.5 to 7.8 m·s^−1^ (ΔPWV −0.7 m·s^−1^) (Mora-Rodriguez et al., 2018). In contrast, a 3-month study reported reductions of ΔPWV −0.5 m·s^−1^ following resistance training and ΔPWV −0.3 m·s^−1^ following HIIT, although the latter did not reach statistical significance (Ramírez-Vélez et al., 2020). More recently, our group demonstrated that 8 weeks of a similar CT_HIIT+RT_ protocol in breast cancer survivors resulted in a greater reduction in arterial stiffness (ΔPWV −1.6 m·s^−1^), despite no significant changes in total body weight (Álvarez et al., 2026). Mechanistically, the beneficial effects of HIIT on arterial stiffness may be explained by increases in retrograde blood flow and shear rate during exercise (Baughman and Sawyer, 2024), as well as enhanced nitric oxide bioavailability (Salmanpour et al., 2022), both of which contribute to improved endothelial function and vascular compliance.

### 4.3 Association between vascular changes with anthropometric/body composition changes after concurrent training

Regarding our third result of associations, delta changes in ΔPWV, ΔFMD, cIMT_av,_ and cIMT_max_ were significantly associated with anthropometric/body composition outcomes ΔWC, ΔBF, ΔSMM, and ΔLM (despite cIMT_av_ with ΔLM) (**Figures 3**, **4**). In a population between 20–59 years old, a descriptive study of (*n* = 10.811) participants reported that abdominal obesity was more closely associated (*r*_s_ = 0.400, *P* < 0.001) with increased PWV than other adiposity stores, such as BMI or subcutaneous fat, showing that individuals with higher PWV also a higher BMI (Wang et al., 2024). In this context, it is well known that changes in body composition, such as lower SMM, worsen cardiovascular health (Zhang et al., 2023) and that previous CT exercise configurations enhance both SMM and various anthropometric/body composition markers (Pedralli et al., 2020; Manojlović et al., 2021; Huang et al., 2023). Our findings align with the ‘fitness and fatness’ topic, where, despite no major weight loss, overweight/obese participants who do not lose weight can still decrease arterial stiffness and increase FMD, as supported by the associations between vascular outcomes and anthropometric/body composition outcomes. Another relevant point to mention is that the primary outcomes expressed in delta changes ΔPWV, ΔFMD, ΔcIMT_av,_ and ΔcIMT_max_ were also associated with secondary anthropometric/vascular outcomes such as ΔSBP, ΔDBP, and ΔMAP, and partially associated with ΔABI with ΔPWV, ΔFMD, and ΔcIMT_max_. Still, only ΔFMD was associated with ΔWeight (**Table 3**). In addition, epidemiological studies by Farrell et al. reported that more than the ‘fatness’ condition (*i.e.* reported by higher BMI), the cardiorespiratory ‘fitness’ outcome was more associated with all-cause mortality in adult women (Farrell et al., 2002b). Following this, we confirmed our hypothesis that CT_HIIT+RT_ would improve vascular ‘function’ independently of weight reduction, which was demonstrated from both improvements reported by the ΔPWV ˗1.2 m·s^-1^ decreases in the EG-NWL (**Figure 2B**) and the increase in ΔFMD +6.9% in the same group with no weight changes (**Figure 2F**). In addition, ΔPWW and ΔFMD were associated with ΔWC, ΔBF %, ΔSMM, andΔLM (**Figure 4**). Future studies should include more complex studies that allow us to elucidate the mechanisms underlying the cause-effect relationship of these results.

### 4.4 Nutritional characterization of the sample

Among other approaches, recent literature from (Lobene et al., 2023) has discussed diet and energy balance and their impact on the vascular function of healthy subjects, where macronutrients and micronutrients could promote better endothelial function in response to exercise. In our current CT program, we applied only exercise, and no diet, however, we described in two times (pre-post) macro-and micronutrients consumption in diet in a sub-sample of (*n* = 9) selected subjects using the 24ASA online questionnaire https://asa24.nci.nih.gov/demo/ (to internal research control) before and after intervention that report in average data (protein: 89.7, fat: 81.3, carbohydrates: 223.5 in average) and micronutrients (Folate: 463.3, Sodium 3473.2, Potassium 2937.3, and Calcium 741). After a 12-week lifestyle intervention (supervised exercise plus diet education) in (*n* = 50) men with prostate cancer with androgen therapy deprivation (Gilbert et al., 2016), reported FMD increases (+2.2%) and endurance capacity by MICT exercise in a treadmill; however, in the present study, we did not apply diet education or a follow-up process after the intervention, where future studies should consider the diet factor as a potential factor for accelerating the exercise adaptations on the cardiovascular health.

### 4.5 Strength and limitations

As limitations, we recognize that *i*) the 6-week exercise period could clearly not have been sufficient to promote greater weight loss effects in participants; however, our approach was to demonstrate that significant weight loss was achieved based on the TE of the measurement used in previous studies of interindividual variability to exercise training in this outcome using the same equipment (Álvarez et al., 2018); *ii*) the FMD procedure was different in some literature studies; for example (Sawyer et al., 2016), used an increase of 250 mmHg of ischemia in the brachial artery during 5 min in this trial, which could limit comparisons with our study in which we used 5o mmHg over the resting SBP value; *iii*) we did not measure cardiorespiratory fitness but measured HR_peak_ during a progressive cardiorespiratory test, *iv*) the menstrual cycle was not considered as a confounding variable in the vascular measurements, *v*) anthropometric measurements were not taken under fasting conditions, and vi) Although we applied the ASA24 dietary questionnaire, its purpose was primarily for dietary ‘characterization’ (aimed at detecting potential changes in diet during a time period) rather than for strict dietary control; however, it was recorded and suggested in the post-tests that a similar breakfast be maintained. Therefore, future studies could consider this aspect to better standardize body composition measurements. However, our study has some strengths: *i*) we used the standard FMD technique with a validated protocol, including the baseline diameter-peak diameter; *ii*) we also used a validated PWV equipment, *iii*) all the participants were under overweight/obesity conditions; *iv*) the present CT program includes both HIIT that promotes oxidative capacity to favor BF oxidation and by contrast RT promotes SMM increases, which could contribute in the future to continuing, maintaining, and improving the initial weight loss results of the participants.

## 5 Conclusion

A 6-week concurrent training program of CT_HIIT+RT_ improved vascular function in overweight and obese women, including those who did not lose weight. Reductions in arterial stiffness and improvements in endothelial function were observed independently of body mass changes and were associated with favorable shifts in body composition and blood pressure (BP). These findings support the value of exercise beyond weight loss in reducing cardiovascular risk. Future trials with longer durations, dietary integration, and direct measures of fitness are warranted to confirm and expand these results.

## Data Availability

The datasets presented in this study can be found in the following online repository https://figshare.com/s/02302db4385062b00293.
